# A New Home Hospital

**Published:** 1887-09-03

**Authors:** Shirley F. Murphy


### A New Home Hospital.

By Shirley F. Murphy, M.R.C.S.

When one of the poorer members of society falls ill, and want of time and the inevitable results of over-crowded centralisation force his friends to abandon the hopeless task of attempting to nurse him, there is one obvious solution of their difficulty—the hospital. Here the poor sufferer can be tended by kind hands, and have the advantage of the best medical and surgical skill in London. But with the richer West-end classes the solution of the like difficulty is not so apparent. A son or a daughter is perhaps laid on the shelf with an attack of typhoid fever. The trouble and inconvenience of nursing the patient soon become evident. Mothers and sisters may be affectionate, but affection is a poor substitute for clinical skill. To send the patient to a hospital, to be visited by his friends only in regulation hours, would be considered little short of barbarous; though undoubtedly, were this course pursued, in the majority of cases the ultimate benefit derived by the patient would amply repay the temporary inconvenience. Still, that the hospitals are little called into requisition by the richer classes is a notorious fact, and some sort of private home, where patients able to afford the luxury of a private room might be nursed through an illness or any operation, has been an obvious want. A bachelor living in rooms rarely wishes to trouble his friends and relations when ill, and it results that in many cases he is nursed by his landlady with more zeal than skill. An officer at the present time invalided home from abroad may be a burden to his friends if nursed at home, and if his relations live in the country he loses the advantage of the advice and attendance of the best London physicians and surgeons. It was to meet this want that, in 1877, Mr. Burdett formulated his successful scheme of Home Hospitals, and the first Home Hospital, Fitzroy House, Fitzroy Square, W., has been almost too popular. The demand upon the available beds is at times overwhelming. Miss Pollock has taken and furnished No. 50, Weymouth Street, Portland Place, which she proposes to devote to Home Hospital purposes. The house is quite new, Miss Pollock being the first occupant. It is well built, and its sanitary arrangements are good. There is a lift, so that invalids may have rooms on any floor without incurring the fatigue of mounting the staircase. The rooms are newly furnished, well lighted, and fitted with Pridgin Teale's grates and Tobin's automatic ventilators. Here, aided by an efficient staff of trained and certificated hospital nurses, Miss Pollock receives patients not suffering from infectious diseases, who can be nursed through illness and attended by their private physicians or surgeons.

The chief advantage of Miss Pollock's Home will be the facility it offers to people in the country. An hotel is not suitable for the reception of a patient awaiting an operation, and in most cases the only alternative offered is to take lodgings, which entails much discomfort and expense. Miss Pollock, who has herself undertaken for many years the duty of a sister at a London hospital, personally superintends the nurses, many of whom she has herself trained. The house has been carefully furnished with a view to the comfort of invalids and convalescents, and all the modern appliances which tend to relieve the sufferer and to make the sick-bed more tolerable are to be found there. In cases where nurses are required at private houses Miss Pollock sends one of her well-trained staff, subject to the ordinary rules, which may be obtained from her.

